# Molecular and epigenetic oncogenesis in synovial sarcoma: implications for cancer biology, diagnosis and treatment

**DOI:** 10.1038/s41388-025-03547-1

**Published:** 2025-08-23

**Authors:** Amy Xueqi Wang, Kevin B. Jones, Torsten O. Nielsen

**Affiliations:** 1 1Department of Interdisciplinary Oncology, Faculty of Medicine, University of British Columbia, Vancouver, BC Canada; 2 Department of Orthopaedics, University of Utah, Salt Lake City, UT USA; 3 Department of Oncological Sciences, Huntsman Cancer Institute, University of Utah, Salt Lake City, UT USA; 4 Department of Pathology and Laboratory Medicine, Faculty of Medicine, University of British Columbia, Vancouver, BC Canada

**Keywords:** Sarcoma, Cancer genetics, Diagnostic markers

## Abstract

The *SS18::SSX* oncogene is the driver of synovial sarcoma, an aggressive cancer presenting in young adults that has poor long-term outcomes. Over the past five years, significant progress has been made in understanding the molecular, genomic, and epigenetic mechanisms underlying synovial sarcoma. This review synthesizes recent advancements in synovial sarcoma, including diagnostic pathology, genomic profiling, SS18::SSX biology, epigenetic dysregulation, proteomics, targetable pathways and immunotherapy. Key findings include the identification of rare but instructive alternative gene fusions, the roles of PRC1 and of liquid-liquid phase separation in SS18::SSX-mediated oncogenesis, and the development of epigenetic and engineered T-cell therapies. These advances offer new hope for improved treatments and outcomes in synovial sarcoma patients, though challenges remain in overcoming resistance and ensuring equitable access to emerging therapies.

## Introduction

Synovial sarcoma (SS) is an aggressive, *SS18::SSX* fusion oncogene-driven cancer typically presenting in periarticular tissues of adolescents and young adults, accounting for about 5% of soft tissue sarcomas. Despite treatment with multimodal therapy, the prognosis for the ~50% of patients who develop metastatic disease remains poor. The standard first-line systemic treatments for metastatic SS, doxorubicin and ifosfamide, achieve partial responses in only ~30% of patients, with 18-month median overall survival. Second-line therapies (including gemcitabine, docetaxel, dacarbazine, eribulin, trabectedin, and pazopanib) offer limited benefit, underscoring the urgent unmet need for novel therapeutic strategies [1].

Recent population-based studies have provided valuable insights into SS epidemiology, treatment patterns, and prognostic factors. The ARST0332 study used a risk-based treatment strategy on 138 newly diagnosed SS patients under 30 years of age. Nearly 1/3 of patients with small, localized tumors could safely avoid chemotherapy and radiation without compromising outcomes, achieving a 5-year overall survival of 100% [2]. However, the study confirmed that metastatic SS continues to have a dismal prognosis, highlighting the critical need for innovative therapies. Analysis of over 2100 SS patient outcomes in the SEER database identified key prognostic factors for localized disease, with age, tumor size, and location significantly influencing outcomes. Patients under 21 years old with small, extremity-based tumors had the lowest risk, while older patients with large, non-extremity tumors (along with those with any metastatic disease) faced the highest risk [3]. Disparities in treatment patterns, such as the preferential use of chemotherapy and radiotherapy in pediatric and wealthier populations, and poorer outcomes among patients with low socioeconomic status and in adults who did not receive radiotherapy, underscore potential confounders in survival analyses and point to the importance of equitable access to care [4].

In the last five years, significant progress has been made in understanding the molecular and cellular biology of SS, paving the way for novel diagnostic and therapeutic approaches. Genomic profiling has uncovered rare alternative fusions that impact our understanding of the key functions of the fusion oncoprotein, and now provides some consensus on the epigenetic dysregulation induced by SS18::SSX. Protein-level studies have implicated RNA splicing and liquid-liquid phase separation in synovial sarcomagenesis. These discoveries have identified potential therapeutic targets, as well as emboldened the advancement of immunotherapy to treat SS.

This review article attempts to synthesize the latest advancements in SS research, including pathology and diagnosis, genomic profiling, epigenetics, proteomics, targetable pathways, and immunotherapy. By integrating insights from population-based studies, molecular biology, and clinical trials, we aim to provide a comprehensive overview of the current understanding of SS biology and highlight promising avenues for future investigation, where important questions remain about the epigenetics, cell-of-origin and microenvironment of this fascinating form of cancer.

## Pathology and diagnostic advances

SS is characterized by distinct molecular and immunohistochemical features with less heterogeneity than most other cancers. Recent advances in pathology have enhanced our understanding of diagnostic tools and of biomarkers for potential origin, providing critical insights for rapid, accurate diagnosis and therapeutic targeting.

### Immunohistochemical markers and diagnostic specificity

The *SS18::SSX* fusion oncogene, resulting from the t(X;18) translocation, remains the hallmark molecular alteration in SS. An SS18::SSX antibody developed in 2020 [5] targeting the neoepitope created at the most common fusion junction (*SS18 exon 10 :: SSX1/2 exon 6*) offers near-perfect specificity and high sensitivity, revolutionizing the IHC-based diagnosis of SS [6]. Eight additional studies [5, 7–13] now report sensitivities ranging from 86 to 100%—a total of 303 positive IHC results from 326 SS samples. One study successfully attributed most negative samples to alternative fusion splice sites (e.g., *SSX exon 4*) that change the junctional epitope [12], while others pointed to technical factors arising from tissue handling and (in some cases) decalcification, highlighting a limitation in certain clinical contexts [7, 9, 13] and an ongoing role for supporting molecular diagnostics.

### Challenges in FISH-based diagnosis

Particularly when SS18::SSX IHC is not available, SS is diagnosed using a combination of histopathology, immunohistochemistry, and molecular demonstration of the pathognomonic t(X;18) DNA or *SS18::SSX* fusion RNA. Interphase fluorescence in situ hybridization (FISH) to detect *SS18* gene rearrangements has been commonly employed as it performs well even on cytology specimens and small formalin-fixed, paraffin-embedded biopsies. Yoshida et al. highlighted the limitations of *SS18* FISH break-apart probes in diagnosing SS [14], where 11 out of 99 SSs tested negative or indeterminate. Of these 11, 3 had *EWSR1::SSX1* fusions (a novel finding with important implications, discussed below), 1 had *MN1::SSX1*, and 1 had *SS18L1::SSX1*; notably, these cases retained SS histology, methylome profiles, and clinical behavior. The remaining 6 showed strong SS18::SSX expression, with 3 confirmed to have *SS18::SSX2* by RNA sequencing. FISH remains a useful tool for molecular confirmation of translocation-associated sarcomas, but as with RT-PCR, is increasingly being supplanted by newer, more comprehensive assays including NGS, nanoString, or methylome profiling [15].

### Cell of origin

While SS has long been recognized as a misnomer as these tumors do not arise from synovium, recent studies have revealed insights into its cell of origin and phenotypic biomarkers. SS often exhibits features of both epithelial and neural differentiation, with De Logu et al. reporting that over 90% express epithelial markers such as cytokeratins and epithelial membrane antigen, as well as stem cell markers CD99 and TRPA1 [16]. 8-93% of cases had focal expression of S100 protein, a marker of neural crest differentiation, while less than 10% expressed SOX10, a transcription factor linked to Schwann cell differentiation. These findings suggest that SS may arise from a cell population with pluripotent epithelial and neural differentiation potential. Another interpretation is that SS18::SSX expression achieves an induced multipotency or pluripotency that reflects the transformation process more than the cell of origin.

Hill et al. provide evidence supporting a mesenchymal stromal cell progenitor for SS [17]. In their mouse model, SS18::SSX expression in a specific fibroblast-like population (Hic1+ Pdgfra+ Lgr5 + ) leads to 100% penetrant SS development, generating tumors closely resembling human SS histologically and molecularly. In this model, SS arises through the stepwise loss of mature fibroblastic features and the reactivation of an embryonic mesenchymal program, identifying this rare fibroblastic subpopulation as a permissive cell of origin. This work not only clarifies the lineage trajectory of SS but also suggests potential biomarkers that could aid in understanding its pathogenesis.

### Perspectives

SS can now be diagnosed quickly and confidently on small biopsies using immunohistochemical and molecular assays commonly available at sarcoma centers. The diagnostic question is largely “solved” at this point, as are the most effective local control measures [18], leaving oncoprotein biology, cellular background and microenvironment, and systemic treatment as the main areas to focus future research in this cancer.

## Genomics

The mechanism of oncogenic reprogramming driving SS has been increasingly elucidated through advances in genome profiling, chromatin dynamics, and epigenetics, which have not only deepened our understanding of SS biology, but also suggest novel therapeutic targeting strategies.

### Genomic profiling

Although SS is characterized by a relatively quiet genome with few recurrent mutations, recent large scale targeted oncogene sequencing studies using MSK-IMPACT (including 74 SSs) and FoundationOne (259 SSs) panels have found that 21% of cases exhibit mutations in chromatin remodelers or histone modifiers, supporting a key role for epigenetic dysregulation in SS pathogenesis [19, 20]. About 7% of SS cases were found to have mutations in *CTNNB1* and 7% in *SETD2*, with some recurrent copy number alterations also identified (losses on chromosomes 3p and 10p).

### Profiling identifies subtypes of SS

Published studies have otherwise largely failed to link histologic subtypes with consistently reproducible underlying genetic, epigenetic, or proteonomic differences. Bulk RNA and DNA sequencing of 91 tumors from 55 patients by Chen et al. describes molecular subtypes of SS with distinct clinical and biological characteristics: subtype I (associated with poor survival, high metastatic potential, and elevated mitotic activity), subtype II (favorable prognosis, fewer metastasis, and monophasic histology) and subtype III (mostly biphasic, mixed responses to neoadjuvant treatments, frequent relapse) [21]. Single-cell RNA sequencing further delineated these subtypes, revealing that subtype I is enriched with mesenchymal cycling cells, subtype II is dominated by endothelial cells, and subtype III shows a high proportion of epithelial cells, which are notably susceptible to chemotherapy [21].

Jerby-Arnon et al. used single-cell RNA sequencing (of 16,872 cells, from 12 human SSs) to identify a “core oncogenic program” expressed in a less differentiated subset of cycling cells [22]. This program was associated with more aggressive tumors and an increased risk of metastasis. SS also showed an “immune exclusion” phenotype, characterized by limited immune infiltration. GeoMX spatial transcriptomics further demonstrated an inverse relationship between the expression of the core oncogenic program and immune cell infiltration, suggesting that immune evasion may play a critical role in SS progression. Of potential therapeutic importance, the study found that combining HDAC and CDK4/6 inhibitors synergistically repressed the core oncogenic program and enhanced T cell-mediated killing in vitro [22].

### Novel SS18 fusions in non-SS tumors

Although *SSX* fusions remain essentially specific to SS (including the aforementioned *SSX* fusions to *EWSR1*, *MN1*, and *SS18L1* found in tumors with SS histology, gene expression and methylomes [14]), targeted RNA and DNA sequencing (using TruSight RNA Fusion, FoundationOne Heme, KAPA Hyperplus, and FusionPlex Pan-Solid Tumor panels) have uncovered novel *SS18* fusions in other neoplasia. These include *MEF2C::SS18* in microsecretory adenocarcinoma of the skin and salivary gland [23], *SS18::NEDD4* in cutaneous spindled and epithelioid sarcoma [24], *SS18::POU5F1* in a primary renal undifferentiated round cell sarcoma [25] and at least 6 cases of *CRTC1::SS18* in undifferentiated round cell sarcomas [26]. These discoveries highlight the apparent versatility of SS18 as an oncogenic driver in a range of malignancies quite different from SS. As SS18 is a known member of two types of mammalian SWI/SNF (BAF) chromatin remodeling complexes—mutations in which contribute to over 20% of all human cancers – its involvement in diverse cancer types is both significant and biologically plausible.

### Perspectives

Data from bulk DNA and RNA sequencing is publicly available on increasing numbers of tumors, although some series are potentially confounded by the effects of neoadjuvant therapy, and links to outcomes may be difficult to translate to contemporary cases undergoing different treatment protocols at other centers. Single-cell methodologies are becoming increasingly employed as their costs decrease. As always, initial discovery-based analyses are hypothesis generating and do require validation on independent series and in functional experiments, ideally by independent groups and in prospective studies. Going forward, AI-based integration of multiple existing datasets and incident data may yield further insights not evident through existing bioinformatic approaches.

## Epigenetics

Epigenetic dysregulation is a key mechanism for SS18::SSX-mediated transformation. SS18::SSX incorporates into BAF chromatin remodeling complexes, leading to aberrant gene activation and tumorigenesis. New studies pinpoint SS18::SSX distribution to hypomethylated promoters with Polycomb repressor complex 1 (PRC1) histone marks. Preclinical studies have shown that some epigenetic drugs can disrupt SS18::SSX-driven transcription, suggesting potential therapeutic avenues.

### Chromatin dynamics and SS18::SSX reprogramming

Hofvander et al’s. methylome and ChIP-Seq for multiple histone marks on primary untreated SS tumor specimens highlights that SS18::SSX creates a continuum of chromatin states, disrupting normal gene expression patterns and promoting tumorigenesis [27]. This reprogramming is mediated by the fusion oncoprotein’s ability (through SS18) to hijack chromatin remodeling complexes, such as CBAF and GBAF, and (through SSX) to recognize altered histone modifications. A striking feature of SS is its unusually hypomethylated genome, particularly at promoter CpG islands, distinguishing it from other sarcomas and most normal tissues [27, 28]. These expanded CpG islands likely recruit KDM2B, a histone demethylase previously identified as a dependency in SS [28]. KDM2B recruits the PRC1.1 complex, which ubiquitinates lysine 119 of histone H2A in regional nucleosomes, which are then recognized by SSX, facilitating SS18::SSX binding [29, 30]. Hofvander et al. also found that the conversion of poised, so-called “bivalent” (H3K4me3 + H3K27me3) gene promoters into actively transcribed genes monovalent for H3K4me3 chromatin marks varied along a continuum in human cases, with those exhibiting more of this reprogramming associated with a better prognosis [27].

A strong affinity for H2AK119ub-decorated nucleosome acidic patches is an intrinsic property of SSX’s carboxy terminus [29–31]. Increased transcription results at developmental genes targeted by SS18::SSX, which distributes GBAF complexes to these hypomethylated promoters with H2AK119ub-marked nucleosomes. The rare SSX fusion oncoproteins with alternative partners (EWSR1::SSX, MN1::SSX) share a similar signature of transcriptionally activated genes to that induced by SS18::SSX1, and also rely on PRC1.1 to instantiate these aberrant gene activation patterns [30]. This conservation of function confirms that SSX is responsible for the localization of SS18::SSX to H2AK119ub-rich regions, even though SSX^1^ does not otherwise demonstrate typical ubiquitin-binding capacity. Tong et al. used the cryo-EM structure of the SSX1 carboxy terminus bound to an H2AK119ub-decorated nucleosome to show that SSX1 binding depends on a cryptic basic groove formed by H3 and the monoubiquitin motif on H2A [31], and in doing so induces DNA unwrapping that likely facilitates transcription factor binding.

SSX increases H2AK119ub by stabilizing PRC1.1 complex in a feed-forward mechanism, underscoring its critical role in mediating specific binding to chromatin [30]. Notably, SS18::SSX is dependent on PRC1 – but not PRC2 – emphasizing the centrality of PRC1.1 in SS oncogenesis [32]. Boulay et al. used patient-derived organoids to demonstrate that SS18::SSX recruits PRC1.1 to deposit H2AK119ub, while opposing PRC2 activity, leading to decreased H3K27me3 [32].

### BAF association

Although the mechanisms driving the genome-wide distribution of SS18::SSX on chromatin are clearly linked to SSX’s affinity for H2AK119ub-decorated nucleosomes in hypomethylated CpG islands in the promoters of developmental genes, the relationship of the other half of the fusion (i.e., SS18, in the vast majority of cases) is a somewhat more complicated area of study [30]. Since not long after the cloning of the genes involved in the t(X;18) translocation, *SS18* has been known to associate with mammalian SWI/SNF (aka BAF) chromatin remodeling complexes.

In 2004, SS tumors were seen by IHC to have a characteristically low, but not absent presence of the BAF47/SMARCB1 protein in their nuclei [33]. In 2012, TAP-tag pull downs identified that SS18::SSX interacts with most of the core BAF subunits, except for those exclusively incorporating into polybromo BAF (PBAF) complexes [34], suggesting that SS18 was indeed a member of BAF complexes. Later that year, a prominent paper claimed that incorporation of SS18::SSX into canonical BAF complexes led to the exclusion of SMARCB1 from those complexes, which resulted in SMARCB1 degradation [35].

What was not yet appreciated was that another BAF complex subtype, later described in 2018 GBAF, represents a SMARCB1-less complex even in its wildtype form, yet also incorporates SS18 (and, in SS, SS18::SSX) [36]. GBAF was also apparent in the original 2012 TAP-tag study, but was not recognized at the time by any name. After the subsequent recognition (from dependency screens) that BRD9, a component GBAF, was a strong dependency in SS cell lines, a controversy began over which complex – GBAF, or CBAF lacking SMARCB1—is incorporating SS18::SSX [37, 38].

**Fig. 1:**
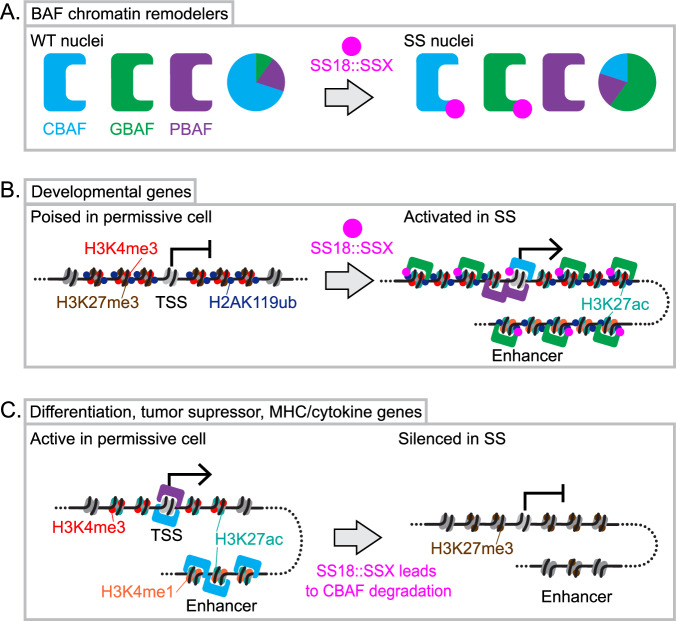
Mechanisms of SS18::SSX-driven oncogenesis in synovial sarcoma. **A** The fusion oncoprotein SS18::SSX avidly binds to chromatin through the interaction of the SSX tail with H2AK119ub, and through SS18 is incorporated into both canonical BAF complexes (CBAF) and GLTSCR1-containing non-canonical BAF complexes (GBAF). This leads to the degradation of CBAF and the relative expansion of GBAF complexes. **B** SS18::SSX thereby recruits GBAF to transcriptional start sites (TSS) in H2AK119Ub-marked chromatin regions. Developmental genes that, in permissive wild type cells, bear bivalently marked (H3K4me3 and H3K27me3) “silenced-but-poised” promoters become converted to an H3K4me3 actively transcribed state in SS. **C** Concurrently, permissive cells rely on CBAF to activate enhancers of differentiation, tumor suppressors, and immune signaling, and loss of CBAF leads to silencing of their transcription, although these loci are less readily identified because they lack H2AK119Ub and are not bound by SS18::SSX.

Studies in a SS mouse model suggest that SMARCB1 is not as completely degraded as had been postulated, prompting further biochemical tests in a recombinant system. These experiments demonstrated that not only could SS18::SSX incorporate into both GBAF and CBAF, but that in the latter, it could cohabitate with SMARCB1 [39]. Subsequent experiments in cell lines confirmed that the partial degradation of SMARCB1 was actually a partial degradation of whole CBAF complexes, where the other components that are CBAF-exclusive (SMARCB1 incorporates into CBAF and PBAF) were even more profoundly depleted in the presence of SS18::SSX (Fig. 1A). Rather than an exclusion of SMARCB1 from BAFs, expression of the fusion oncoprotein drives degradation of a major portion of canonical BAF complexes in the SS nucleus [39].

### DNA methylation

Koelsche et al. developed a DNA methylations classifier for sarcomas, trained on 1077 tumors and validated on 428 samples. This publicly available dataset provides a robust tool for molecular classification of sarcomas, including SS with its highly distinct methylome, and may aid in identifying epigenetic subtypes with therapeutic implications [28]. Genome-wide methylation analyses comparing primary to recurrent/metastatic tumors showed similar methylation levels, but differentially methylated regions, associating these epigenetic changes with disease progression [40]. Array comparative genomic hybridization showed no significant chromosomal gains or losses between the primary tumor and metastasis, further implicating epigenetic rather than genomic alterations in SS progression [40].

### Perspectives

**Fig. 2:**
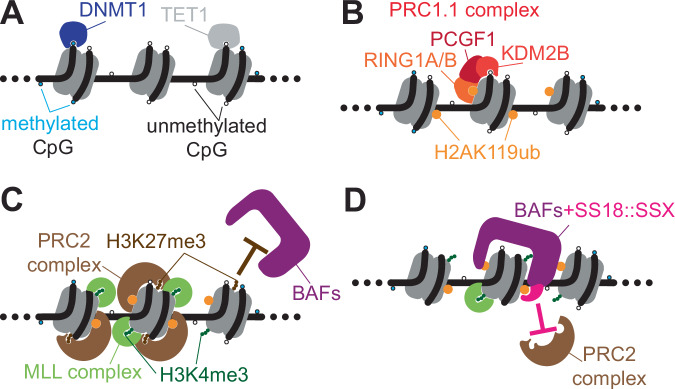
Epigenetic readers and writers pertinent to the distribution of SS18::SSX. **A** CpG islands are typically hypomethylated at the promoters of developmental genes, for which hypomethylation is established and maintained by TET1 demethylase (upregulated in synovial sarcoma). DNMT1, which methylates CpG islands, is down-regulated in most synovial sarcomas, but not lost entirely. **B** The non-canonical Polycomb repressive complex PRC1.1 recognizes hypomethylated CpG islands through its KDM2B subunit, and monoubiquitylates lysine 119 of histone H2A—a pre-repressive post-translational modification to regional nucleosomes recognized by SSX. **C** In normal cells, the H2AK119ub mark is read by the JARID2 component of the PRC2 complex, which then trimethylates lysine 27 of histone H3. These nucleosomes are termed bivalently marked, as lysine 4 in histone H3 is also trimethylated by MLL complexes (which have WDR5 as a core component). This H3K4me3 mark is considered an active mark, but when accompanied by H3K27me3 in bivalency will yield a poised, but not transcribed developmental gene, because H3K27me3 is thought to impede the activity of BAF chromatin remodeling complexes that could otherwise open the chromatin for expression. **D** In the presence of the fusion oncoprotein SS18::SSX, BAF complexes incorporating the fusion are avidly recruited to nucleosomes bearing the H2AK119ub mark, rendering PRC2 unable to trimethylate H3K27, and activating transcription from the promoter’s gene.

The oncobiology of SS involves SS18::SSX-induced reprogramming of an epigenetically-determined cell state (Fig. 1). Hence, in the absence of drugs directly targeting SS18::SSX, epigenetic drug strategies provide the best hope to reverse that process. Since 2020, there have been significant advances in our understanding of the epigenetic mechanisms underlying SS – abnormal methylation and histone modification patterns, and the role of PRC1 and BAF complexes in maintaining the SS oncogenic program (Fig. 2). Future research should expand on epigenomic profiling (more histone marks; use of single cell and spatially-defined techniques) and on developing and testing more epigenetic drugs, including exploring combination therapies that may increase efficacy.

## Proteomics and phase separation

### Proteomic signatures and RNA splicing pathways

A next-generation proteomics method, SWATH (sequential window acquisition of all theoretical fragment ion spectra) mass spectrometry, was used to analyze formalin-fixed specimens from 36 soft tissue sarcomas including 7 SSs [41], identifying 103 proteins overexpressed in SS. The most significant category consisted of 24 proteins that have roles in mRNA splicing regulation, including pre-mRNA splicing proteins (SRSF1, SRSF3, SRSF7, SRSF9, SRSF11), heterogeneous ribonucleoprotein particle (hnRNPs) proteins, and spliceosome A Complex proteins (HNRNPA1, U2AF2, SNRNP70). These findings suggest that enrichment of RNA splicing and processing pathways could play a role in SS pathogenesis.

Tang et al. subsequently used proteomic and phosphoproteomic data to evaluate 272 FFPE soft tissue sarcoma samples, including 18 SS, and 91 matched tumor-adjacent tissues [42]. Using cell-type deconvolution analyses, SS showed elevated Th1 lymphocyte signatures linked to poor outcomes. Additionally, a conserved molecular signature was observed among SS, rhabdomyosarcoma, leiomyosarcoma, dedifferentiated liposarcoma, and undifferentiated sarcoma, where these patient samples showed increased proliferation, APEX1, and NPM1 abundance. SS most closely clustered with rhabdomyosarcoma, both displaying enrichment in RNA processing and metabolism pathways, including tRNA processing, RNA degradation, and spliceosome components.

The proteomics landscape of SS was further elucidated in 2023 by Burns et al. using Tandem Mass Tag 11-plex mass spectrometry on 321 soft tissue sarcoma FFPE specimens, including 43 SSs [43]. Although no specific ontologies were enriched in SS, there was an upregulation of proteins involved in non-homologous end joining, including PRKDC, XRCC1, XRCC5, XRCC6, RAD50 and MRE11. This enrichment of double-strand break repair proteins did not differ between pre-operatively treated and non-treated patients. Unlike the studies above, Burns et al. did not identify enrichment of RNA processing or spliceosome-associated proteins, possibly due to their use of Tandem mass spectrometry as SWATH-MS is more sensitive to some low-abundance proteins (like RNA splicing factors). Nevertheless, the identification of elevated NHEJ proteins, regardless of treatment status, suggests a potential role for DNA double-strand break repair in SS biology that warrants further exploration.

### Phase separation and oncogenic activity

In 2021, Kuang et al. showed that wildtype SS18 regulates a pluripotent to somatic transition through liquid-liquid phase separation [44]. A CRISPR-based screen identified SS18 and BRG^1^ (aka SMARCA4), both subunits of BAF complexes, to be top hits. Immunostaining showed that SS18 forms punctate condensates, facilitated by its intrinsically disordered domain in C-terminal amino acids 223-379. The SS18 N-terminal interacts with BAF and is required for the transition.

Subsequently, Cheng et al. used X-ray crystallography to resolve the structures of human SS18/BRG1 and yeast SNF11/SNF2 [45]. These subcomplexes showed comparable conformations, suggesting that SNF11 is a yeast homolog of human SS18. More importantly, the tyrosine residues in the intrinsically disordered QPGY domain of SS18 (which are retained in SS18::SSX) were identified as key for mediating liquid-liquid phase separation and the subsequent recruitment of BRG1.

Kuang et al. (2024) then used immunofluorescence imaging, FRAP, in situ Hi-C, and CUT&Tag to show that both native SS18 and oncogenic SS18::SSX1 proteins can form nuclear condensates [46]. SS18::SSX1 condensates specifically exclude histone deacetylases HDAC1 and HDAC2. This leads to an abnormal accumulation of histone H3 lysine 27 acetylation (H3K27ac), along with CBP/p300 at specific chromatin sites, activating oncogenic genes Forcing HDAC1 into SS18::SSX condensates via N12(Sall4)-NuRD reduces activation of downstream genes like PAX6 and NKX3-2. Additionally, this change reduces cell proliferation, colony formation, and tumorigenesis in xenografts. Furthermore, selective CBP/p300 catalytic inhibition with A-485 decreases H3K27ac modified histones in SS18::SSX condensates. These findings suggest that the remodeling of nuclear condensates by SS18::SSX1 disrupts normal gene regulation, promoting tumorigenesis, and highlights potential therapeutic targets within these altered condensates.

Corroborating these findings, Li et al. used a small molecule screen and immunofluorescence imaging to show that SS18::SSX increases the number of protein condensates compared to SS18 [47]. These condensates are also more resistant to 1,6-hexanediol, and are more thermodynamically stable. When H2AK119ub histone modification is inhibited with PRT4165, nuclear condensates diffuse significantly, SS18::SSX downstream genes (including *SOX2*, *PAX7*, and *NKX3-2*) are no longer activated, and cell proliferation is reduced. Thus, this activity of SS18::SSX is dependent upon H2AK119ub recognition by the C-terminal domain of SSX. Additionally, SS18::SSX1 condensates capture and subsume SS18 condensates, changing the localization of SS18 and impeding the expression of SS18-targeted tumor suppressor genes, much akin to the CBAF loss mechanism discussed above. This competition between different condensates adds a new dimension to determining cell fate.

### Perspectives

Structural and other protein-level studies converge on a picture where the primary contribution of the SS18 part of the fusion oncoprotein is to confer phase separation capacity by forming molecular condensates with chromatin, which alter transcription and RNA processing.

## Targetable pathways and novel therapeutics

Recent advances in SS biology have identified several targetable pathways, offering new therapeutic opportunities. These include epigenetic modifiers that are biological dependencies in SS, transcription factors, cyclin-dependent kinases, apoptotic mechanisms, growth factor signaling, redox homeostasis pathways and cell surface targets.

### Epigenetic biological dependencies

As SS18::SSX-driven sarcomagenesis relies on epigenetic modifications, DNA demethylating drugs like decitabine and azacytidine—already approved for myeloproliferative disorders—are promising therapies. In SS, they phenocopy the effect of DNA methyltransferase DNMT1 knockdown, inducing global hypomethylation. This results in de-repression of many mesenchymal differentiation genes, growth arrest, and production of extracellular matrix. These drugs showed efficacy against all six tested SS cell lines, a cell line xenograft, and a genetic mouse model [48]. Although oral formulations of both agents have recently been developed, neither has yet been tested in SS clinical trials.

Two groups have highlighted the potential use of a small molecule inhibitor of SAE1/2, called TAK-981, in SS [49, 50]. SAE1/2, the E1 enzyme that launches the SUMOylation cascade, modulates chromatin‑remodeling proteins often co‑opted in cancer; in synovial sarcoma its activity supports tumor survival and proliferation, with DepMap and other datasets flagging several SUMO‑pathway genes as essential dependencies [49, 50]. One of these studies has mechanistically dissected the specific impact of TAK-981 and found that it restores CBAF levels even in the presence of SS18::SSX [49]. Although TAK-981 seems promising, in November 2024 its Phase I/II clinical trial (NCT03648372) ended early after termination of its drug development program by the trial’s sponsor.

Another SS dependency, BRD9, was targeted by multiple companies to bring BRD9-degrading PROTACs to phase I clinical trials. The trial for FHD-609 [51] demonstrated on-target degradation of BRD9 in patients (in on-treatment tumor tissue biopsies), but disappointing clinical activity (only one partial response in 55 patients), as well as worrisome cardiac electrophysiology side effects that have put further development of this and a similar agent (CFT8634: NCT05355753) on hold.

DepMap has also identified USP7, a deubiquitinase, as a SS dependency. USP7 stabilizes PRC1.1, and its depletion reduces proliferation in SS cell lines, making it a promising therapeutic target, further supported by *USP7* knockdown studies in patient-derived organoids [32].

Similarly, WDR5 was shown by CRISPR screening to be another dependency in SS. Yu et al. showed WDR5 physically associates with SS18::SSX-containing BAF complexes, co-localizing genome-wide at promoters and enhancers of developmental oncogenes such as *MNX1* and *SOX8* [52]. Degradation of WDR5 eliminates ~70% of its chromatin peaks, suppresses H3K4me2/3, and concomitantly reduces the occupancy of SS18::SSX and BAF155 without altering H2AK119ub, indicating that WDR5 helps “lock in” the fusion-BAF transcriptional program. Therapeutically, twice-daily injection of the WDR5 targeting-PROTAC, MS67, shrinks HSSY-II xenografts and prolongs mouse survival, degrades WDR5 and RBBP5 in tumors and down-modulates *FZD10*, *SOX8*, and ribosomal protein coding-genes [52].

### Transcription factors

CREB, activated by SS18::SSX is critical for SS survival; CREB inhibition with 666-15 suppresses SS cell proliferation and induces apoptosis in vitro and in vivo [53]. These results suggest CREB inhibitors (666-15, KG-501, and NASTRp) and the PKC-inhibitor Ro 31-8220 (PKC acts upstream of CREB) are potential clinical trial candidates.

ETS transcription factors ETV4 and ETV5 have been identified as key downstream effectors of SS18::SSX, driving SS progression through the regulation of cell cycle pathways and the DUX4 embryonic program. ETV4 and ETV5 activation promotes proliferation, survival, and metastatic potential in SS cells [54]. Targeting these transcription factors or their upstream regulators represents a potential strategy for disrupting SS oncogenesis, although effective drugs to achieve this are not yet available.

### Cyclin-dependent kinase inhibitors and their combination with histone deacetylase inhibitors

CDK7 has been found to be highly expressed in SS, linked to worse outcomes [55]. The CDK7 inhibitor BS-181 demonstrated activity in vitro, albeit at high IC50 values, suggesting it may need further optimization [55]. Similarly, CDK4 is highly expressed in SS, and is associated with worse prognosis [56]. A phase II clinical trial of the CDK4/6 inhibitor palbociclib as a single agent in advanced sarcoma patients, including two with SS, produced a median PFS and overall survival of 4.2 and 12 months, respectively [57].

Following up on their identification of a core oncogenic program in SS that included CDK4, Jerby-Arnon et al. found that CDK4/6 inhibitors acted synergistically with histone deacetylase (HDAC) inhibitors in SS [22]. The combination of HDAC and CDK4/6 inhibitors repressed the core oncogenic program, enhanced SS immunogenicity, and induced T cell reactivity and T cell-mediated killing [22]. Cooley and Su highlighted the role of HDAC inhibitors in acetylating MDM2, subsequently leading to the degradation of SS18::SSX thru the MULE ubiquitin ligase—thereby providing a histone-independent mechanism of action for HDAC inhibitors in SS [58]. Indeed, HDACs are likely misnamed as they function more generally as protein deacetylases, meaning HDAC inhibitors hit many targets beyond histones. Lanzi et al. report that HDAC inhibition activates the ERK-EGR1-heparanase survival pathway, which can be counteracted by combining HDAC inhibitors with trametinib, a MEK inhibitor [59]. This combination showed synergistic effects in SS cell lines and xenograft models.

### Cell cycle and apoptosis pathways

Wang et al. demonstrated that S-phase kinase-associated protein 2 (SKP2) overexpression plays an essential role in SS oncogenesis [60]. Depletion of SKP2 reduced proliferation in SS cell lines and inhibited xenograft tumor growth. The SKP2 inhibitor flavokawain A induced cell cycle arrest and apoptosis, highlighting SKP2 as a potential therapeutic target.

Although SS is known to express high levels of the antiapoptotic protein BCL-2 [61], it appears resistant to the BCL-2 inhibitor venetoclax [62]. MCL-1 may provide an alternative protection against apoptosis in SS; Fairchild et al. [63] showed that targeting MCL-1 (either through expression of the MCL-1 inhibitor NOXA or treatment with the BH3 mimetic S63845) sensitizes SS cells to venetoclax [63]. In PDX models, the combination of venetoclax and S63845 induced tumor regression, suggesting a promising therapeutic strategy. A phase 1 trial combining venetoclax with S64315, a close analog of S63845, was recently completed in AML [63], raising the possibility of its subsequent testing in SS once dosing and toxicities are reported, although trials of similar drugs showed dose-limiting toxicities related to cardiovascular function [64].

### Growth factor signaling

DeSalvo et al. identified FGFR1/2/3 as critical for SS tumor growth. In SS mouse models, FGFR knockout or inhibition with BGJ398 suppressed tumor growth and downstream ERK signaling, leading to reduced expression of ETV4/5, transcription factors essential for SS progression [54]. FGFR inhibition also upregulated DUX4, an embryonic pathway gene, inducing SS cell death.

Brahmi et al. found that platelet-derived growth factor alpha, and its receptor PDGFRA, is highly expressed in SS [65]. However, the anti-PDGFRA antibody olaratumab did not improve outcomes in a trial for advanced soft tissue sarcomas [66], suggesting that alternative strategies targeting this pathway may be needed, a dedicated SS trial needs to be designed, or that expression of PDGFA and its receptor may be more a marker of the cell-of-origin phenotype of SS [17] than of an important functioning driver of its neoplastic growth.

BRAF V600E mutations have been reported in three patients with thoracic synovial sarcoma, two of whom demonstrated transient clinical responses to BRAF/MEK inhibitors [67, 68]. Unfortunately these targetable mutations appear to be very rare in sarcomas, with clinical-grade panel sequencing of 259 synovial sarcomas failing to reveal any bearing BRAF V600E mutations [20].

### Redox homeostasis and ferroptosis

Malic enzyme 1 is frequently absent in SS, shifting antioxidant dependence from the glutathione system to the thioredoxin system. This makes SS cells more susceptible to redox stress and ferroptosis. Cystine transport inhibitors ACXT-3102 and erastin induce lipid peroxidation and ferroptosis in SS models, a novel therapeutic approach [69].

### Cell surface targets

Molecules expressed on the cell surface can be targeted with antibodies conjugated to radionuclides or to cytotoxic drugs. Frizzled homolog 10 (FZD10) is a cell surface receptor involved in Wnt signaling that is overexpressed in most SS [70]. A phase 1 trial of β-radioimmunotherapy with the ^90^Y-labeled anti-FZD10 antibody OTSA101 produced a best response of stable disease in 3 of 8 patients, lasting up to 21 weeks [71]. Subsequent work developed OTSA101 labeled with the α-emitter ^225^Ac, which at least in SS cell line xenograft models achieved complete remissions in 3 out of 5 mice [72].

Antibody-Drug Conjugates are increasingly being developed, following their success in breast cancer. Geller et al. evaluated the antibody-drug conjugate lorvotuzumab mertansine (IMGN901), which targets CD56, a marker expressed in SS amongst other tumor types. In a phase 2 trial involving 62 pediatric patients (including 10 with SS), 3 SS patients achieved stable disease lasting 6 cycles, and 1 achieved a delayed complete response. These results suggest that CD56-targeted therapy may benefit a subset of SS patients [73].

### Perspectives

In the absence of drugs that directly target the SS18::SSX oncoprotein, we are left having to target its partners, downstream effectors, and their induced oncogenic pathways and surface markers. These strategies will be less specific and more toxic than a direct inhibitor of SS18:SSX, but would likely have applications in other types of cancer – opening opportunities for drug repurposing strategies, faster-accruing trials, and greater interest from pharmaceutical companies.

## Immunotherapy and immune evasion

SS is considered an “immune cold” tumor, by virtue of its low mutational burden and lack of immune infiltrates. However, its reactivation of embryologic programs leads to production of proteins that can be recognized and targeted by the immune system. Additionally, biphasic SS exhibits higher levels of immune cell infiltration in the spindle cell compartment compared to monophasic SS, implying differences between these subtypes that may influence immunotherapy responses [74].

### Cancer-testis antigens

SS is a promising candidate for some forms of immunotherapy due to its frequent expression of cancer-testis antigens, such as PRAME, NY-ESO-1, and MAGE-A4. PRAME, a repressor of retinoic acid receptor-mediated differentiation and apoptosis, is expressed in 70% of SS cases (152/216), although it has also been detected at a high frequency in some other malignancies, including myxoid liposarcomas, some carcinomas, and melanomas [75]. A recent sarcoma-focused immunohistochemical study confirmed 100% PRAME expression in 11 SS cases – most at the highest level of interpretable staining intensity, matched only by myxoid liposarcoma, embryonal rhabdomyosarcoma and intimal sarcoma [76]. MAGE-A4 and NY-ESO-1 are also frequently expressed in SS, with a recent study of 91 SS cases studies reporting positive IHC staining 69% and 56% of cases, respectively [77]. Notably, NY-ESO-1 expression was higher in metastatic lesions compared to primary tumors, suggesting an association with disease progression.

PRAME has recently emerged as a target for immunotherapy in SS. Trials evaluating T-cells targeting HLA-restricted PRAME peptides have demonstrated promising anti-tumor activity in PRAME-positive solid tumors, including SS: in the Phase 1b ACTengine IMA203 trial, all three SS patients achieved tumor shrinkage, including one objective response [78]. Additional trials, such as NCT03686124 and NCT04262466, are exploring engineered T-cell receptor therapies targeting PRAME in SS and other cancers. Recent preclinical studies show that SS18::SSX drives PRAME expression, which has a role in modulating retinoic acid signaling; indeed, combination treatments incorporating all-trans retinoic acid lead to reduced proliferation and induction of cellular senescence in SS models [79].

NY-ESO-1 and MAGE-A4 are clinically-advanced immunotherapy targets in SS. NY-ESO-1 has been extensively studied, with a phase I/II trial (NCI 08-C-0201) demonstrating a 50% objective response rate in SS patients treated with autologous T-cells expressing engineered NY-ESO-1-specific TCRs. However, high rates of adverse events tied to cytoablative regimens highlight the need for careful patient selection and management [80]. NY-ESO-1 was recently found by immunohistochemistry to be expressed in only 56% of SS samples, with significantly higher expression in metastatic lesions [77]. These findings underscore the importance of biomarker-based patient stratification. In parallel, ongoing trials such as NCT01343043 and NCT03967223 aim to optimize TCR affinity and reduce toxicity while improving response durability.

Clinical trials NCT03132922 and NCT04044768 are evaluating T-cell therapies targeting MAGE-A4 in solid tumors, including SS, and have provided evidence of anti-tumor activity in patients with high antigen expression. Afamitresgene autoleucel, a genetically engineered T-cell therapy targeting MAGE-A4 in the context of one of four subtypes of HLA*02, represents a new option for SS systemic therapy in advanced disease. The SPEARHEAD-1 phase 2 trial [81] demonstrated efficacy in heavily pretreated, advanced SS (43% objective response rate; median duration of response 6 months), leading to FDA approval for unresectable or metastatic SS expressing MAGE-A4 in an appropriate HLA context. This marks the first adoptive cell therapy approved for any solid tumor, underscoring the potential of immunotherapies in SS (and the lack of effective alternatives).

### Immune checkpoint inhibitors

Despite the success of immune checkpoint inhibitors in other cancers, they have no apparent efficacy in SS consistent with this tumor’s immune-excluded microenvironment (NCT03132922, NCT04044768, NCT03686124, NCT04262466, NCT01343043, NCT03967223, NCT04526509, NCT04939701) [81–83]. However, combination strategies are still being explored, pairing ICIs with tyrosine kinase inhibitors or chemotherapy that may enhance immune infiltration and anti-tumor activity. Ongoing trials in mixed soft tissue sarcoma cohorts, including SS, are investigating such combinations, with results pending (NCT03307616, NCT03474094, NCT04028063) [84, 85].

### Perspectives

Given the biology and oncogenesis of SS, it is not surprising that the most successful forms of immunotherapy (i.e., checkpoint inhibitors targeting PD[L]1) have failed to benefit SS patients. However, the heavy expression of multiple cancer-testis antigens does create an opportunity for immune augmentation strategies based on engineered T-cells, leading to at least one new approved treatment for advanced metastatic SS—a situation where other agents provide little if any benefit. However, these strategies cost several hundred thousand dollars per patient, involve highly toxic induction therapies, require an HLA type present in <20% of the population (and particularly uncommon among ethnic minorities), and result in often not durable responses in less than half of patients. Also, none of these approaches targets the SS18::SSX oncoprotein.

## Conclusion

SS remains a complex and difficult malignancy, but the last 5 years have brought game changing advancements in understanding its biology. This review highlights key breakthroughs in diverse areas, including diagnostics, genomics, epigenetics, proteomics, targetable pathways, and immunotherapy, that have collectively enhanced our knowledge on this aggressive sarcoma of adolescents and young adults. The ongoing development of targeted therapies, such as demethylases, antibody-drug conjugates, and engineered immune cells, offers the promise of treatment advances. Nevertheless, challenges remain in translating preclinical findings into the clinic due to recruitment limitations and sometimes unforeseen adverse drug effects of new agents.

The future of SS research and treatment is poised for continued innovation. Diagnosis and local control measures are effective and well advanced, but major gaps persist in our knowledge of SS biology (how SS18::SSX acts on the epigenome in tumor transformation and evolution, the requisite permissive cellular background, and the mechanisms generating an immunoevasive microenvironment). Key areas for research include insights through genomic and epigenomic sequencing, and development of drugs targeting SS18::SSX and its associated pathways and dependencies. By building on these advancements, the field is well-positioned to address these remaining challenges and thereby improve outcomes for those affected by this often devastating disease.
